# Powassan Virus Encephalitis, Minnesota, USA

**DOI:** 10.3201/eid1810.120621

**Published:** 2012-10

**Authors:** Justin Birge, Steven Sonnesyn

**Affiliations:** Abbott Northwestern Hospital, Minneapolis, Minnesota, USA

**Keywords:** Powassan virus, encephalitis, fatal, tick, viruses, vector-borne infections, Minnesota

## Abstract

Powassan virus (POWV) is a rare tick-borne agent of encephalitis in North America. Historically, confirmed cases occurred mainly in the northeastern United States. Since 2008, confirmed cases in Minnesota and Wisconsin have increased. We report a fatal case of POWV encephalitis in Minnesota. POWV infection should be suspected in tick-exposed patients with viral encephalitis.

## Case Report

A 67-year-old woman from Aitkin, Minnesota, USA, sought treatment at a local hospital on May 30, 2011, with a 3-day history of dizziness, fever of up to 103°F (39.4°C), chills, malaise, nausea, and occasional confusion with slurred speech. She had no respiratory or gastrointestinal symptoms and no history of ill contacts, travel, environmental exposures, or other recent illness. She had not been exposed to animals or vectors, other than those endemic to her area of residence, which included mosquitoes and deer ticks. She had removed many deer ticks after gardening or hiking in the woods. The patient’s past medical history was notable for colon cancer, which had been treated with a partial colectomy in October 2010 and chemotherapy through April 2011. She also had a history of hypertension, cutaneous lupus, and a remote cerebral aneurysm with surgical clipping. Medications she was taking were atenolol, hydroxychloroquine, and valsartan.

On admission, the woman was alert and reported mild neck tenderness. Her temperature was 100°F (37.8°C), blood pressure 138/77 mm Hg, pulse rate 83 beats/min, respiratory rate 22 breaths/min, and oxygen saturation 98% on room air. Results of neurologic, cardiac, and respiratory examinations were normal. Studies with normal test results included comprehensive metabolic panel, urinalysis, computed tomographic scan of the head, and chest radiograph. Results of serum screen for *Borrelia burgdorferi* antibodies (by ELISA) were negative. Leukocyte count was within reference range (10.8 × 10^3^/mm^3^), with neutrophil (polymorphonuclear leukocytes) predominance (80%). Her cerebrospinal fluid (CSF) showed 80 leukocytes (89% polymorphonuclear leukocytes), 5 erythrocytes, and 64 mg/dL of protein. The patient was given piperacillin/tazobactam and doxycycline.

The next day, she was less responsive and was transferred to Abbott Northwestern Hospital in Minneapolis. Shortly thereafter, she became unresponsive and labored breathing developed. Her temperature reached 102°F (38.9°C), and the following laboratory values were outside the reference range: leukocyte count (11.3 × 10^3^/mm^3^), sodium level (131 mmol/L), erythrocyte sedimentation rate (49 mm/h), and protein level (2.3 mg/dL). Neurology and infectious disease specialists suspected viral encephalitis. Magnetic resonance imaging (MRI) was deferred because of the unknown composition of the aneurysm clip, and the patient underwent a computed tomography angiogram of the head and neck. Infarction, vasculitis, meningeal enhancement, and structural abnormalities were not found. Twenty-four–hour electroencephalogram monitoring and administration of ceftriaxone (2 g intravenously [IV] every 24 h), acyclovir (500 mg IV every 8 h), and doxycycline (100 mg IV every 12 h) were initiated.

Overnight, the patient became apneic and required intubation. Examination revealed absent deep tendon reflexes, ocular deviation, positive Babinski response, and bilateral flaccid paralysis of the extremities. Pupillary light and corneal reflexes remained intact. No independent respirations were initiated. Complement levels were within reference range. No evidence of seizure was shown on electroencephalogram, although epileptiform discharges were seen. Given the severity of encephalopathy, prophylactic levetiracetam was initiated.

**Figure 1:**
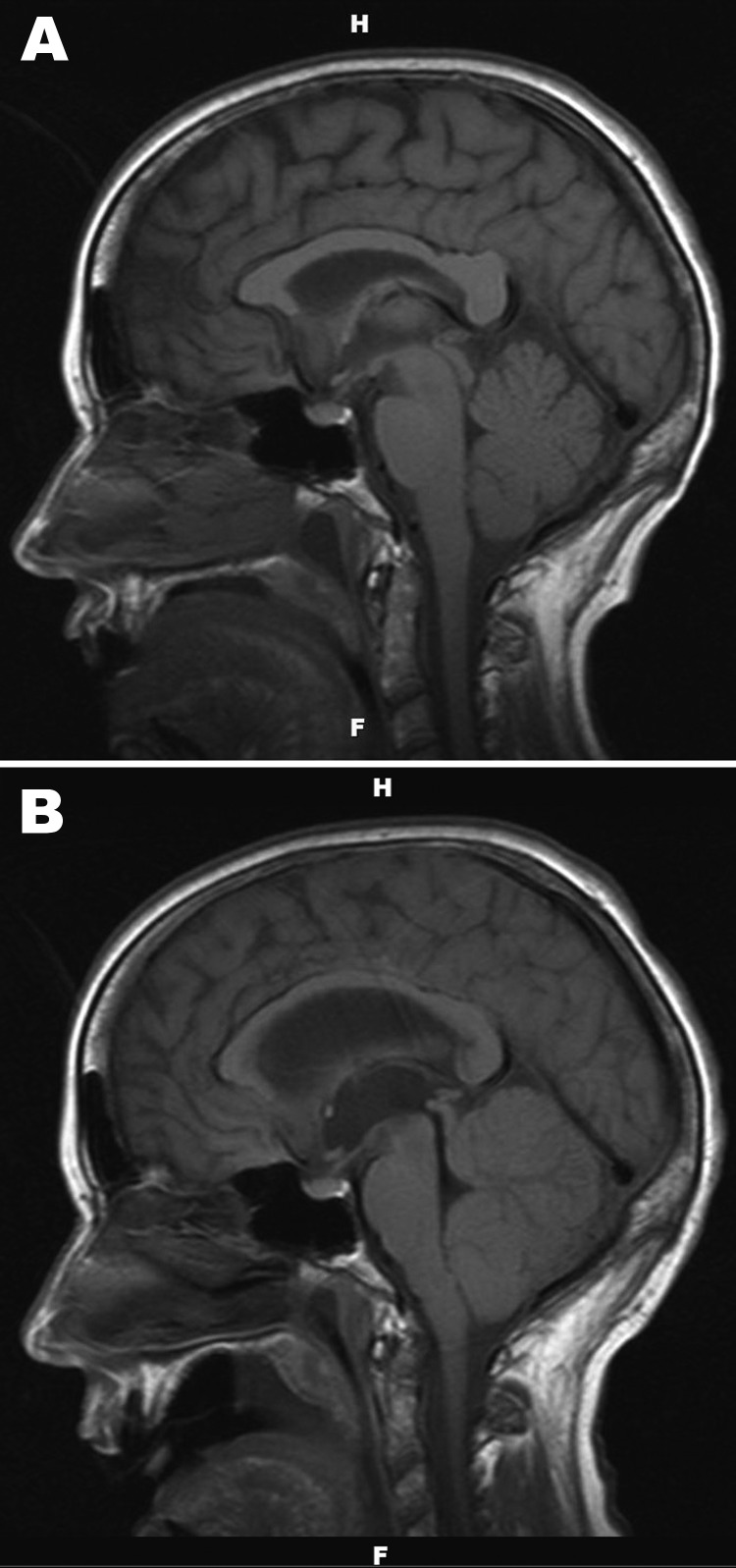
A) Noncontrast, sagittal T1-weighted magnetic resonance image of the brain of a 67-year-old woman with suspected Powassan virus encephalitis, obtained 4 days after admission. Image is notable for nonspecific signal changes within the thalami, midbrain, cerebellar vermis, and both cerebellar hemispheres. B) Noncontrast, sagittal T1-weighted magnetic resonance image of the brain obtained 8 days after patient’s admission. Changes include marked interval progression of signal abnormality involving the cerebellum, thalamus, and midbrain. Mass effect within the posterior fossa and crowding of structures at the foramen magnum are also evident. Marked dilatation of the lateral and third ventricles with acute hydrocephalus is apparent.

Results of repeat screen for *B. burgdorferi* antibodies, smear for *Ehrlichia* spp., and blood and urine cultures were unremarkable. Brain MRI revealed nonspecific inflammatory changes within the thalamus, midbrain, and cerebellum, with no evidence of meningeal irritation, temporal lobe abnormality, mass effect, acute infarct, or hydrocephalus (Figure 1, panel A). Acyclovir was discontinued. Routine bacterial culture of CSF was negative. Ceftriaxone and doxycycline were continued because acute Lyme disease, which rarely manifests in this manner, could not be ruled out by serologic testing alone.

The patient remained unresponsive with flaccid paralysis and areflexia. Four days after the initial examination, repeat MRI showed substantial progression of signal abnormality in cerebral hemispheres, thalamus, and midbrain. Mass effect was evident with crowding of structures at the foramen magnum. Lateral and third ventricle dilation, consistent with acute hydrocephalus, was noted (Figure 1, panel B). A repeat lumbar puncture was not performed, given clinical interpretation of illness, known imaging findings, key pending results, and lack of indications for additional testing.

Thirteen days after illness onset, a serology panel was negative for the following viruses: West Nile, La Crosse, Eastern equine encephalitis, St. Louis encephalitis, and Western equine encephalitis. PCR of CSF was negative for enteroviruses and herpesviruses. The Minnesota Department of Health (MDH) reported positive IgM serologic results against Powassan virus (POWV). Ceftriaxone and doxycycline were discontinued. Given the patient’s clinical deterioration and poor prognosis, she was electively extubated and then died. The Centers for Disease Control and Prevention confirmed POWV infection by IgM serology and reverse transcription PCR of CSF.

**Figure 2:**
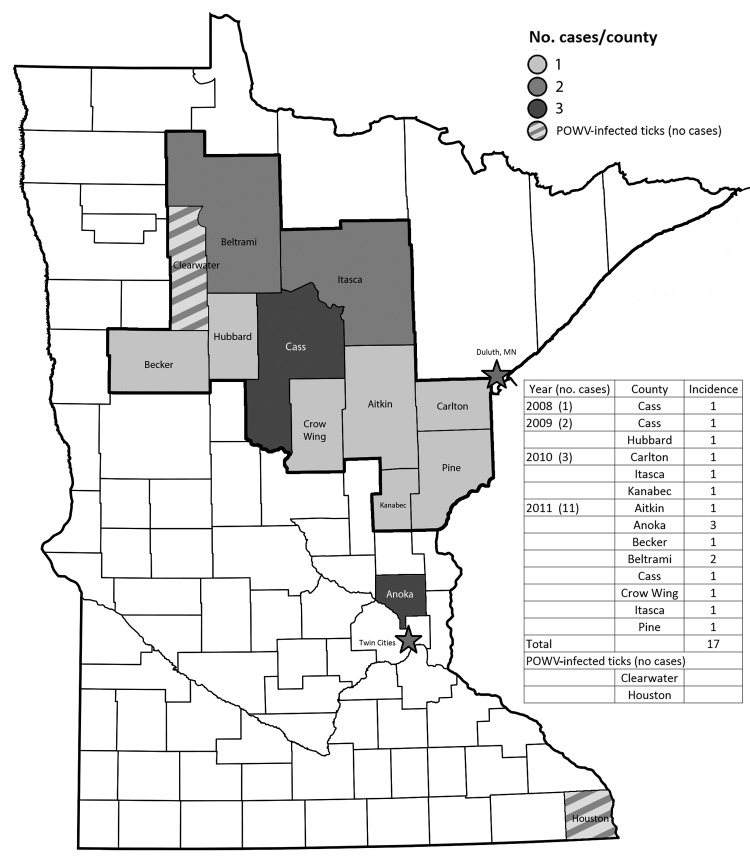
Geographic distribution of confirmed Powassan virus (POWV) infections (diagnosis made by serology, reverse transcription PCR) and counties with POWV–infected ticks in Minnesota. Data provided by the Minnesota Department of Health.

POWV is a tick-borne member of the family *Flaviviridae,* first reported in 1958 (*1*). It is the only tick-borne member of the genus *Flavivirus* with human pathogenicity in North America (*2*). Selection bias in identifying the infection may exist, diminishing the reported incidence to only patients with severe disease. Results of seroprevalence studies in Canada and the northeastern United States are variable but include seroprevalence estimates as high as 5.8% in Canada (*3*) and 0.7% in New York State (*4*). Small and medium-sized mammals are common reservoirs (notably, woodchucks [*Marmota monax*] and white-footed mice [*Peromyscus leucopus*]), and several species of tick (4 *Ixodes* spp*.*, 2 *Dermacentor* spp.) act as vectors (*5*–*7*). Human infection has been documented in North America and Russia (*8*). Both prototypic (POWV) and deer tick virus (DTV) genotypes exist (*6*,*9*). In Canada and the northeastern United States, *I. cookei* ticks are the typical vectors for POWV. In Minnesota and Wisconsin, *I. scapularis* ticks are the typical vectors for DTV. Although *I. cookei* ticks are present in these states, they rarely attach to humans, and according to MDH data, all sequenced strains in Minnesota are of the DTV genotype. Transmission time from tick to host has been documented in mice as quickly as 15 minutes (*10*). The length of attachment time required for disease-causing viremia in humans is unknown. Literature estimates vary, but <40 cases were documented during 1958–2000 (*2*,*4*,*11*,*12*). According to the Centers for Disease Control and Prevention and the health departments of Minnesota and Wisconsin, 33 US cases were reported during 2001–2010: 12 in New York, 9 in Wisconsin, 8 in Minnesota, 2 in Maine, and 1 each in Michigan and Virginia. In 2011, of 16 confirmed cases in the United States, 11 occurred in Minnesota and 4 occurred in Wisconsin. Figure 2 illustrates the geographic distribution of human cases and infected ticks.

Patients with POWV infection typically exhibit encephalitis after an incubation period of 1–4 weeks. Fever and headache are common; the typical viral prodrome lasts 1–3 days. Mental status changes, cerebellar symptoms, and hemiplegia are also common and may be severe. Results of CSF testing and brain imaging are generally consistent with viral encephalitis. Reverse transcription PCR of CSF, serologic testing of CSF, and serologic testing of serum are the preferred diagnostic tests, but they are not widely available. Diagnostic testing for POWV should be referred to state and federal laboratories to ensure accuracy and standardization (*4*,*8**,**9**,**11*–*13*)

Pathogenesis is due to lymphocytic infiltration of perivascular neuronal tissue with a predilection for gray matter, including thalamus, midbrain, and cerebellum (*11*). Supportive care is the only therapy. A European vaccine against the related tick-borne encephalitis virus is available, but although the viruses are antigenically similar, its effectiveness against POWV is unknown (*12*). Approximately 10% of the reported infections have been fatal, and an additional 50% have produced long-term neurologic sequelae, including hemiplegia and headaches (*2*,*9*,*13*).

## Conclusions

POWV is causing an emerging and potentially severe tick-borne infection in Minnesota and Wisconsin. POWV infection should be suspected when tick-exposed patients exhibit viral encephalitis, especially those with cerebellar symptoms and/or thalamus/midbrain gray matter disease. Preventing tick attachment by using chemical prophylaxis and vigilance are essential in disease-endemic environments to prevent contraction of POWV infection.
